# Classification Terminology and Definitions in Reporting of MRI in Axial Spondyloarthritis

**DOI:** 10.5334/jbr-btr.1393

**Published:** 2017-12-16

**Authors:** Danishta Ramdin, Arumugam Moorthy, Winston J. Rennie

**Affiliations:** 1 Leicester Royal Infirmary, UK

**Keywords:** MRI, Seronegative spondyloarthropathy, Spine, sacroiliac joint, radiology, reporting, diagnosis

## Abstract

Spondyloarthritis (SpA) is a group of chronic inflammatory conditions which severely impact quality of life. Several criteria have been developed in the past to aid the diagnosis of SpA based on symptoms and radiographic changes during the course of the disease. However, it takes several years before structural changes manifest on conventional radiographs, leading to a diagnostic delay of 6 to 10 years. The use of MRI and its incorporation into the Assessment of Spondyloarthritis (ASAS) criteria, has radically changed the diagnosis of SpA in the last decade by allowing visualisation of both active and chronic inflammatory changes and enabling clinicians to recognise SpA during it’s early stage and initiate treatment. An understanding of the various terminology used in the divisions of disease presentations and their relevant imaging findings are key, along with the use of clear definitions of structural and inflammatory changes on MRI, in ensuring accurate diagnosis and classification of SpA.

## Introduction

Spondyloarthritis (SpA) is a spectrum of immune-mediated chronic inflammatory changes and encompasses a variety of rheumatic diseases such as ankylosing spondylitis, psoriatic arthritis, reactive arthritis and entheropatic arthritis. SpA affects about 1% of the population [1], men and women equally, in their late twenties. It is strongly associated with HLA-B27 which is present in about 90% of patients diagnosed with SpA [2]. SpA can broadly be divided into axial SpA (ax-SpA) and peripheral SpA depending on the involvement of the spine and axial skeleton.

## Clinical Presentation

The main presentation of ax-SpA is back pain with evidence of inflammation. Both axial and peripheral SpA are associated with extra-articular manifestation such as uveitis, colitis and skin lesions. Peripheral Spa is characterised mainly by inflammation in the limbs, enthesis and dactylics without symptoms of low back pain.

Based on the ASAS criteria, SpA can be broadly diagnosed by a clinical and imaging arm. Whereby the clinical arm is defined by positive HLA-B27, the imaging arm is the manifestation of active sacroilitis on MRI [3]. With the use of MRI, a sub-group of SpA has been identified as non-radiographic SpA (nr-SpA) [4]. Radiographic SpA refers to patients in the clinical or imaging arm with evidence of sacroiilitis on X-rays of the Sacro-iliac joint (SIJ), whereas non-radiographic SpA can be defined as inflammatory back pain in the absence of structural damage on conventional radiographs. The percentage of nr-SpA subjects is believed to be as high as 80% in the Imaging arm and 20% in the clinical arm. The progression of nr-SpA to the end stage of radiographic ankylosis is, as yet, undetermined. Table 1 summarises the ASAS classification and the varying definitions of classification criteria.

**Table 1:**
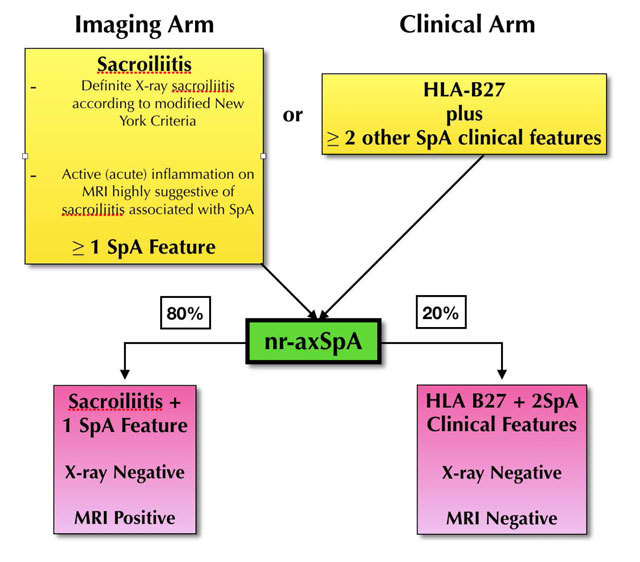
Summary of ASAS Criteria and nr-SpA [10].

Forty percent of patients with non-radiographic SpA have signs of spinal inflammation on MRI [5]. Studies have shown that 10–20% of patients with non-radiographic SpA progress to ankylosing spondylitis (AS) over two years [6]. Recently there has been a debate about the significance of treatment of non-radiographic SpA due to the small percentage of people who progress to AS and the cost of treating with anti-TNF or DMARDs and its efficacy [7].

There are no specific diagnostic tests for SpA; it is diagnosed based on clinical symptoms and radiographic changes of sacroiliac joints. There has been a diagnostic delay of around 5–10 years [8] in the past, due to the non-specific clinical presentation of SpA and lack of radiographic changes on radiographs of the SIJ during the initial stage of the disease. The advent of biologic drugs has allowed an improved quality of life in this condition and treatment. Over the past 20 years several diagnostic criteria have emerged allowing identification of a larger cohort of patients, and more recently (including cases of non-radiographic SpA), with the advent of ASAS criteria. The recent use of MRI in SpA and its inclusion in the ASAS criteria has allowed clinicians to diagnose this condition early and optimise clinical intervention [9].

## Clinical Features

The primary clinical features in ax-SpA are inflammatory low back pain, limited chest expansion and buttock or hip pain. Table 2 summarises the clinical features assessed as part of the ESSG and Modified New York criteria.

**Table 2:** Clinical features as part of ESSG criteria [17].

Variable	Definition

Inflammatory spinal pain*	History or present symptoms of spinal pain in back, dorsal, or cervical region, with at least four of the following: (a) onset before age 45, (b) insidious onset, (c) improved by exercise, (d) associated with morning stiffness, (e) at least three months duration.
Synovitis	Past or present asymmetric arthritis or arthritis predominantly in the lower limbs.
Family history	Presence in first-degree or second-degree relatives of any of the following: (a) ankylosing spondylitis, (b) psoriasis, (c) acute uveitis, (d) reactive arthritis, (e) inflammatory bowel disease.
Psoriasis	Past or present psoriasis diagnosed by a doctor.
Inflammatory bowel disease	Past or present Crohn disease or ulcerative colitis diagnosed by a doctor and confirmed by radiographic examination or endoscopy.
Alternating buttock pain	Past or present pain alternating between the right and left gluteal regions.
Enthesopathy	Past or present spontaneous pain or tenderness at examination at the site of the insertion of the Achilles tendon or plantar fascia.
Acute diarrhoea	Episode of diarrhoea occurring within 1 month before arthritis.
Urethritis/cervicitis	Non-gonococcal urethritis or cervicitis occurring within one month before arthritis.
Sacroiliitis	Bilateral grade 2–4 or unilateral grade 3–4, according to the following radiographic grading system: 0 = normal, 1 = possible, 2 = minimal, 3 = moderate and 4 = ankylosis.

**The Modified New York Criteria (MNY) [11]**

Clinical criteria:

- Low back pain and stiffness for more than three months that improves with exercise, but is not received by rest.
- Limitation of motion of the lumbar spine in the sagittal and frontal planes.
- Limitation of chest expansion relative to normal values correlated for age and sex.

Radiological criteria:

- Sacroilitis grade > or = 2 bilaterally or 3–5 unilaterally.

The modified New York criteria was the first diagnostic tool specific to include radiographic changes of the sacroiliac joints in ankylosing spondylitis; this was divided into clinical and radiological criteria. AS was diagnosed if at least one clinical criterion was present along with radiological sacroiliitis; however, it does not include aspects of peripheral SpA and sacroilitis on radiograph as mandatory criteria. The MNY criteria was shown to have specificity but low sensitivity of 16.9% [2].

## MRI in Ax-SpA

MRI has had a huge impact in the diagnosis and management of SpA. MRI allows diagnosis of SpA during the initial stages of the disease and is able to demonstrate features of both acute inflammatory stages and chronic stages. Prior to the use of MRI in SpA, efficacy of treatment was measured based on patient related symptoms only. The course of the disease can be monitored, based on changes seen on MRI. Compared to conventional radiography with a sensitivity of 19% and specificity of 47% in detecting active sacroilitis, MRI had a sensitivity and specificity of 95 and 100%, respectively. MRI has also allowed SpA to be diagnosed in 75% of patients missed by conventional radiographs [12,13,14]. In one study, MRI allowed early diagnosis of sacroilitis in 39.5% patients without radiological changes as well as ruling out active lesions in 60.4% of cases with sacroilitis changes on conventional radiographs based on the modified New York criteria [15].

The advantage of MRI is that it does not use ionising radiation. However, it is more expensive than conventional radiographs, is less available and may involve longer imaging times. MRI Protocols can be based on the recent recommendations, published by the ESSR arthritis subcommittee and essentially involves imaging of the Spine and SIJ [16].

## MRI Acquisition

Slice positioning and angulation are critical for accurate assessment of the SIJ. The SIJ is a complex joint which is not in a standard orthogonal plane. Semi coronal or semi-axial planes have to be utilised for accurate assessment of the joint with a view to avoid the partial volume phenomenon of the fibrous portion of the joint. T1-weighted images (T1-W) and STIR images are sufficient in most adult cases to assess for stigmata of SpA.

In the Spine, sagittal T1-W and STIR images with an adequate extension of slices to include the costo-transverse joints and beyond will allow accurate assessment of disease burden. Some studies have suggested that at least 20% of thoracic inflammatory disease maybe missed if standard spinal MRI from pedicle to pedicle is performed [18].

## Discussion

Active inflammation includes bone marrow oedema (BMO), capsulitis, enthesitis and synovitis. Other lesions such as erosions, sclerosis, Fat infiltration and ankylosis represent sequelae of inflammation and may broadly be considered as structural lesions or non-active lesions. In the radiological diagnosis of the disease, a holistic approach should be utilised with a systematic assessment of anatomical sites within the SIJ and the number and presence of structural lesions on T1-W imaging primarily. The presence of bone marrow oedema on STIR or T2 Fat suppressed imaging, suggests active inflammation, and increases diagnostic confidence.

The creep of classification criteria into the radiological diagnostic process can sometimes lead to over analysis and under interpretation of imaging findings, especially in subtle and early cases. It remains to be seen, with improved clinical diagnosis, how much of an impact this will have on patient populations, if the true rates of progression to full blown ankylosis are, as yet, unknown in nr-SpA.

Based on the current ASAS criteria, to diagnose active sacroilitis, it is essential for bone marrow oedema or osteitis to be present on STIR sequences. One active lesion must be seen on two consecutive slices or more than one lesion on one slice, must be present. Presence of bone marrow oedema is essential for the diagnosis of active sacroilitis; the presence of other lesions such as synovitis, enthesis or capsulitis marrow oedema without the presence of BMO/osteitis is debatable and cannot be classified as active inflammation. These criteria have recently been updated with the inclusion of more radiologists on the ASAS consensus panel [19]. Recent studies have shown that administration of contrast is not recommended due to the cost, risks of side effects and prolonged scanning time [3,14,20,21,22].

Findings in the spine and SIJ on MRI using T1 and STIR sequences, can be broadly divided into structural and inflammatory lesions. Tables 3 and 4 categorise common structural and inflammatory lesions in the spine and SIJ, respectively. The varying terminology and definitions are categorised to aid in reporting.

**Table 3:** Typical Structural and Inflammatory Lesions on Spinal Imaging.

Corner Inflammatory Lesion (CIL)	This presents as bone marrow oedema and appears as a triangular or L shape in one quadrant of the vertebra, commonly along the anterior or posterior margin on mid sagittal imaging. Related to the entheses of the anterior and posterior longitudinal ligaments with the annulus fibrosis and the cerebral body.
Central Inflammatory Lesion	Andersson lesion, typically appears as a semi-circular area of bone marrow oedema, related to the vertebral end plate adjacent to the intervertebral discs and can be associated with erosions.
Costotransverse Joint Inflammation (CTJ)	Adjacent bone marrow oedema on the far lateral sagittal images, related to the junction of the rib and the transverse process of the adjacent thoracic vertebra. Absent at T11 and T12.
Costovertebral Joint inflammation (CVJ)	Can affect any joint from T1 to T12. Circular pattern of bone marrow oedema related to the posterior intervertebral disc and middle column of the vertebral body. It can extend to the adjacent soft tissue, rib margin and posterior aspect of vertebral bodies.
Enthesitis of spinal ligaments supraspinous ligament and interspinal ligaments	Supraspinous, interspinous ligament inflammation, seen along the spinous processes in the mid sagittal slices, along the posterior elements.
Syndesmophytes/ankylosis	Manifests as a linear continuous marrow signal between vertebral bodies on MRI. May occur on para sagittal slices and not on the central sagittal imaging.

**Table 4:** Typical Structural and Inflammatory Lesions in the SIJ.

Erosions	Most varied in presentation. Visible on T1 images as loss of cortical bone associated with adjacent low bone marrow signal intensity. If active, manifests as a hyper intense lesion or with extensive adjacent bone marrow oedema on STIR. (Figure 2)
Fat Infiltration	Can be difficult to diagnose in young active adults with patchy marrow fat. If at least two these criteria are followed, may be easier to diagnose accurately. Juxta-articular – in contact with the articular surface. (Figure 1) Geographical – sharp margins Signal – Uniform marrow signal intensity on T1-W images.
Sclerosis	Uniform Low signal intensity on T1 and STIR imaging, in the subchondral region. (Figure 3)
Ankylosis	Continuous marrow signal intensity across the joint. Can also manifest as marrow across parallel sclerotic tram-track lines believed to be residual joint lines from previous long erosive changes affecting the SIJ.

**Figure 1:**
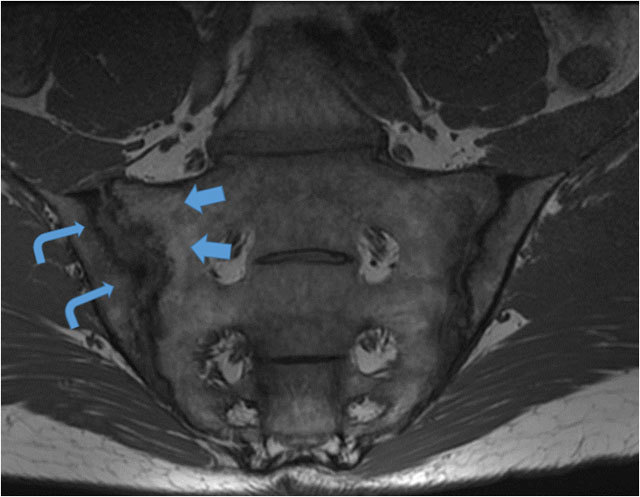
T1-W mid coronal oblique image through the SIJ in a 42-year-old male who presented with long standing low back pain, raised inflammatory markers and a positive HLAB27. Juxta-articular geographical fat infiltration (blue arrows) is noted as structural lesions, along the sacral margin of the SIJ. Pseudowidening of the joint on MRI with large elongated erosions along the iliac aspect and subarticular sclerosis (curved arrowheads) of the right SIJ.

**Figure 2:**
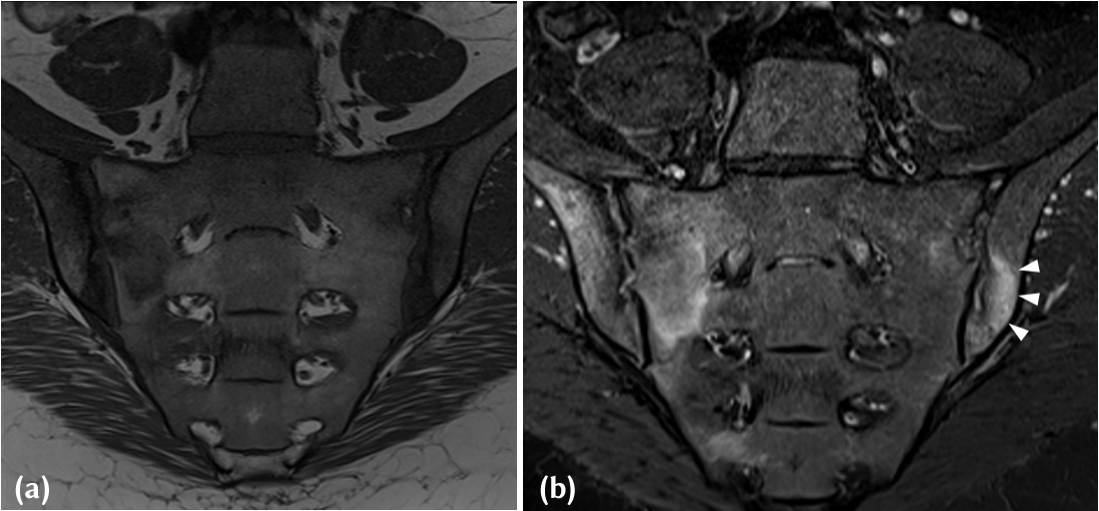
**(a)** T1-W Mid-coronal MRI in a 38-year-old HLAB27 positive, Female patient who presented with inflammatory back pain and buttock pain. Areas of joint space widening with iliac sided erosions and adjacent marrow low signal intensity are noted in keeping with active inflammatory lesions. **(b)** Corresponding STIR image in a 38-year-old HLAB27 positive, Female patient who presented with inflammatory back pain and buttock pain, demonstrates extensive high signal intensity (white arrowheads) in the adjacent subchondral region and marrow in keeping with inflammation associated with an active erosion.

**Figure 3:**
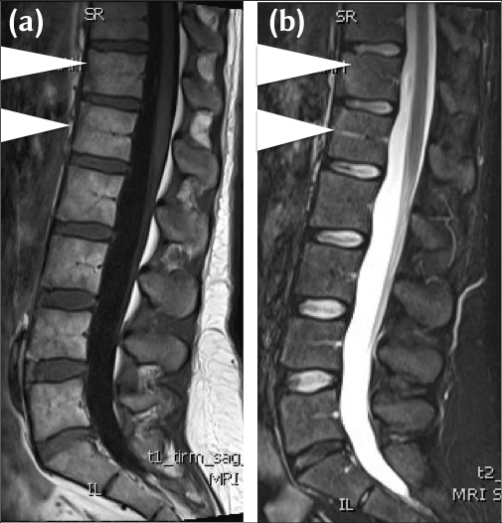
**(a)** 32-year-old female HLA b27 positive, with chronic low back pain and early morning stiffness. Mid sagittal T1-W MRI demonstrates triangular areas of heterogenous intermediate to low signal intensity to fat (between white arrowheads), vertebral corner lesions at T12 and LI. The features are of sclerosis and a Romanus’ Lesion on MRI. **(b)** 32-year-old female HLA b27 positive, with chronic low back pain and early morning stiffness. Mid sagittal STIR MRI demonstrates corresponding triangular areas of low signal intensity (between arrowheads) at T12/L1. The features are of sclerosis and a Romanus’ Lesion on MRI.

## Conclusion

The use of MRI in SpA has revolutionised the definition of SpA, allowing a better understanding of the course of this condition and quantifying responses to treatment. Understanding of the terminology utilised in commonly used rheumatology classification systems and of the division of the disease (nr-SpA), allows a clinician centred reporting strategy for radiologists. Clear radiological definitions and terminology used in ax-SpA are discussed in this review.
